# Alkaline Phosphatase to Albumin Ratio as a Novel Predictor of All-Cause Mortality in Critically Ill Patients With Atrial Fibrillation

**DOI:** 10.1155/crp/1283547

**Published:** 2025-11-04

**Authors:** Haosheng Wu, Xueqian Shen, Yu Xin, Xue Jiang, Caixia Guo

**Affiliations:** Cardiovascular Center, Beijing Tongren Hospital, Capital Medical University, Beijing 100730, China

**Keywords:** albumin, alkaline phosphatase, all-cause mortality, atrial fibrillation, prognosis

## Abstract

**Background:**

Alkaline phosphatase to albumin ratio (APAR) is an emerging prognostic indicator for sepsis, cancer, and coronary artery disease. However, the predictive value of APAR in patients with atrial fibrillation (AF) has not been investigated yet. Therefore, this study aims to explore the association between APAR and the risk of mortality in critically ill patients with AF.

**Methods:**

The data of AF patients were extracted from the Medical Information Mart for Intensive Care-IV (MIMIC-IV) database. Patients with AF were divided into three groups according to the APAR tertiles. Study outcomes were defined as 28-day and 365-day all-cause mortality. The Kaplan–Meier analysis was conducted to compare the survival rates between groups. Cox proportional hazards regression and restricted cubic spline (RCS) were used to investigate the association between APAR and all-cause mortality. Receiver operating characteristic (ROC) curve analysis was utilized to evaluate the predictive value of APAR for study outcomes.

**Results:**

A total of 1105 critically ill patients with AF were enrolled in the study. The Kaplan–Meier analysis demonstrated that patients with the highest APAR had the lowest survival rate. The Cox regression analysis indicated that the highest APAR tertile was significantly associated with 28-day (HR, 1.64 [95% CI 1.20–2.25]; *p*=0.002) and 365-day (HR, 1.87 [95% CI 1.47–2.39]; *p* < 0.001) all-cause mortality. Nonlinear relationships between APAR and 28-day and 365-day all-cause mortality were illustrated based on the RCS curves. The areas under the ROC curves for predicting 28-day and 365-day all-cause mortality were 0.617 and 0.642, respectively.

**Conclusions:**

Our research suggested that APAR was a simple biomarker for the prognosis in patients with AF.

## 1. Introduction

Atrial fibrillation (AF), a prevalent sustained arrhythmia, affects approximately 33.5 million individuals globally [1–4]. It is reported that the estimated spending related to AF in the United States was about 28.4 billion in health care, making the management and treatment of AF a key clinical concern [5]. The sudden decrease in atrial systole and rapid ventricular rate leads to higher mortality rates in patients with severe AF. Data suggested that the incidence of AF in the intensive care unit (ICU) was 15.6%, and patients with AF exhibit higher rates of thromboembolism, bleeding events, and mortality [6]. Hence, the accurate evaluation and risk assessment of critically ill patients with AF is crucial in facilitating clinicians to make the best treatment choices.

The Sequential Organ Failure Assessment (SOFA) score, a widely used tool for monitoring ICU patients' status and predicting mortality risk, has been validated for its prognostic value in critically ill patients with AF [7]. However, the SOFA score, while comprehensive, is overly complex and involves numerous indicators. Assessing the degree of dysfunction in multiple organ systems can be time-consuming, and the predictive capability of the SOFA score for long-term prognosis in cardiovascular diseases is limited [8]. Therefore, there is an urgent need to identify a simple indicator to assist doctors in stratifying the risk of mortality in critically ill AF patients.

Alkaline phosphatase (ALP) is a membrane-bound enzyme that catalyzes the hydrolysis of organic pyrophosphate at basic pH values [9]. Elevated ALP levels are associated with various pathological conditions, particularly those related to bone and liver disease. Recent evidence implicated the potential role of ALP in prompting vascular calcification, thereby contributing to the progression of atherosclerosis [10]. Moreover, higher ALP levels were significantly related to poor cardiovascular prognosis in AF [11]. Albumin is synthesized in the liver and plays a protective role in cardiovascular diseases by exerting anti-inflammatory, anti-oxidative stress, anti-platelet aggregation, and anticoagulant effects [12]. Serum albumin was associated with the recurrence of AF following pulmonary vein isolation ablation [13]. ALP to albumin ratio (APAR) was an easily accessible indicator for adverse outcomes in patients with sepsis [14], cancer [15], and coronary artery disease [16]. Additionally, APAR exerted better prognostic value than the SOFA score in patients with sepsis [14]. However, the relationship between APAR and clinical outcomes in patients with AF remains unknown.

This study aimed to investigate the potential association between APAR and all-cause mortality at 28 days and 365 days in critically ill patients with AF using the Medical Information Mart for Intensive Care-IV (MIMIC-IV) Version 2.2 database.

## 2. Methods

### 2.1. Database and Study Design

This retrospective observational study utilized data from the MIMIC-IV (Version 2.2) database, encompassing clinical records of ICU stays at Beth Israel Deaconess Medical Center (BIDMC) from 2008 to 2019. Over 70,000 ICU admissions and comprehensive information of patients, including demographics, life signs, laboratory examination, treatment, and prognostic data, were recorded in the MIMIC-IV database. Since the database is publicly available for application, ethical approval and informed consent are not required. One author accomplished learning assignments and obtained access to the database (certification number: 59614630).

### 2.2. Data Extraction

Data in the present study were extracted by Navicat Premium (Version 16.1.15) utilizing structured query language (SQL): (1) demographics: age, male, race, body mass index (BMI). (2) Vital signs: systolic blood pressure (SBP), diastolic blood pressure (DBP), mean blood pressure (MBP), heart rate (HR), respiratory rate (RR), and temperature. (3) Laboratory tests were collected within 24 h after admission, including C-reactive protein, white blood cell (WBC), red blood cell (RBC), platelet, hemoglobin, prothrombin time (PT), partial prothrombin time (PTT), international normalized ratio (INR), partial pressure of O_2_ (PO_2_), alanine aminotransferase (ALT), aspartate aminotransferase (AST), creatinine, blood urea nitrogen (BUN), sodium, potassium, albumin, lactate, glucose, anion gap, base excess (BE), triglyceride, cholesterol, and ALP. (4) Score system: SOFA score. (5) Comorbidities incorporated myocardial infarction, heart failure, peripheral vascular disease (PVD), cerebrovascular disease (CVD), hypertension, diabetes, and chronic kidney disease (CKD). (6) Medications: aspirin, clopidogrel, statins, amiodarone, calcium channel blockers (CCBs), angiotensin-converting enzyme inhibitors or angiotensin receptor blockers (ACEI/ARB), warfarin, and nonvitamin K antagonist oral anticoagulants (NOACs). The study outcomes were all-cause mortality at 28 days and 365 days.

### 2.3. Study Population and Outcomes

A total of 12,137 patients diagnosed with AF admitted into ICU for the first time were collected. AF patients were defined according to the International Classification of Diseases codes, incorporating 42,731, I48, I4811, I4820, I4821, I4819, I481, I4891, I482, I480, and I489. The exclusion criteria were as indicated below: (1) patients aged < 18 years; (2) patients who stayed in ICU less than 24 h; (3) patients who lacked a record of ALP, albumin, weight, height, PO_2_, and MBP within 24 h of ICU admission; and (4) patients identified with malignant cancer (ICD: 140–172, 1740–1958, 200–208, 2386, C43, C88, C00–C26, C30–C34, C37–C41, C45–C58, C60–C76, C81–C85, C90–C97), metastatic solid tumor (ICD: 196–199, C77–C80), acquired immune deficiency syndrome (ICD: 042, 043, 044, B20, B21, B22, B24), and severe liver diseases (ICD: 4560, 4561, 4562, 5722–5728, I850, I859, I864, I982, K704, K711, K721, K729, K765, K766, K767). The final cohort of the study comprised 1105 critically ill patients with AF. The study endpoint was defined as the all-cause mortality rate at 28 days and 365 days following ICU admission.

### 2.4. Statistical Analysis

In this study, variables with more than 20% missing data were excluded, while those with less than 20% missing data were handled using Multiple Imputation by Chained Equations (MICE). The number of imputations was set to 5 with 5 iterations, and a fixed random seed was applied to ensure reproducibility. Categorical variables (including outcome variables) did not have missing data and were not included in the imputation model. For example, C-reactive protein, triglyceride, and cholesterol had missing rates exceeding 20% and were excluded (Table S1). All of the continuous variables were non-normally distributed (analyzed by the Kolmogorov–Smirnov test) and expressed as median (interquartile range [IQR]). Categorical variables were presented as frequency (percentage). For the comparison of continuous variables, one-way ANOVAs or the Kruskal–Wallis test were applied based on the normality of the variables. Chi-squared or Fisher's exact test was used to compare the categorical variables between groups. According to the tertile range of APAR on admission, individuals were divided into three groups. Kaplan–Meier survival analysis and the log-rank test were utilized to analyze the mortality during follow-up between the APAR-based groups. Three Cox proportional hazard models were employed to determine the hazard ratio (HR) and the 95% confidence interval (CI) for APAR and all-cause mortality. The variance inflation factor (VIF) was calculated to assess the multicollinearity among variables (Table S2). Variables with a VIF higher than 5 were excluded. Model 1 was unadjusted. Model 2 incorporated age, sex, race, and BMI. Given the number of mortality events available, Model 3 involved parameters in Model 2 and was further adjusted for variables that were selected based on a two-step strategy. First, we prespecified a set of variables based on their established association with mortality in critically ill patients or AF pathophysiology, as reported in prior literature [17–20]. Second, to avoid overfitting given the number of events, we applied a stringent statistical filter (*p* < 0.01 in univariate analysis) to identify the most potent variables from this predefined set for inclusion into Model 3. Therefore, Model 3 was adjusted for male, age, race, BMI, heart failure, CVD, diabetes, CKD, BUN, lactate, potassium, SOFA, HR, ACEI/ARB, warfarin, and NOACs. APAR was involved in the models with both continuous and categorical variables. To validate the robustness of missing data handling, sensitivity analyses (complete-case data) were conducted for the fully adjusted Model 3. The lowest tertile of APAR was regarded as the reference in the three models. The restricted cubic splines (RCSs) adjusted for confounders in Model 3 were drawn to illustrate the association between APAR and all-cause mortality. The receiver operating characteristic (ROC) analysis was applied to determine the predictive ability of ALP, albumin, APAR, and SOFA score for 28-day and 365-day mortality in critically ill patients with AF, and the area under the curve (AUC) was evaluated. DeLong's test was conducted to compare the AUC differences between APAR, ALP, albumin, and SOFA score. In addition, subgroup and interaction analyses were conducted based on age (< 65 or ≥ 65), sex (male or female), hypertension, diabetes, myocardial infarction, and heart failure to evaluate the prognostic value of APAR in different subsets of individuals. The Spearman's correlation analysis was performed to explore the association between APAR and clinical markers (including WBC, ALT, AST, and creatinine). All analysis was performed by SPSS software (Version 22.0) along with R software (Version 4.2.2), and a two-sided *p* < 0.05 was deemed statistically significant.

## 3. Results

### 3.1. Individual Characteristics

**Figure 1:**
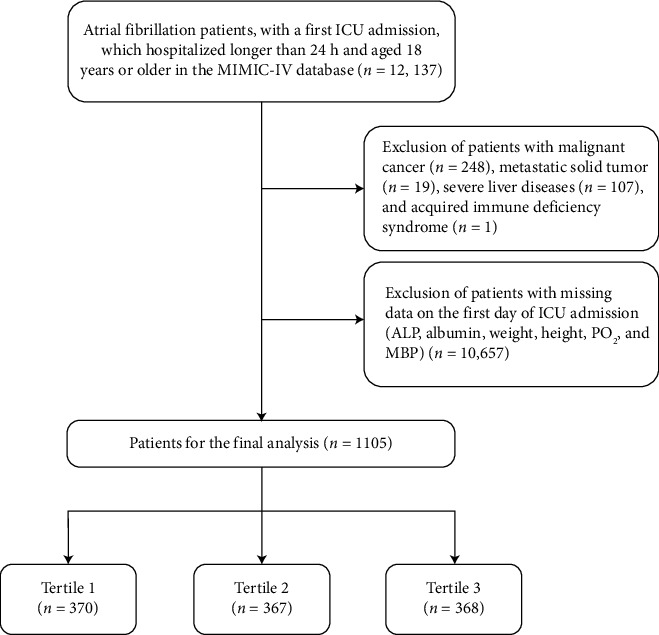
Flowchart of the study. ALP, alkaline phosphatase; MBP, mean blood pressure; PO_2_, partial pressure of O_2_.

A total of 12,137 AF patients over 18 years old, admitted to the ICU for the first time and stayed in the ICU for more than 24 h, were incorporated in the present study. Finally, 1105 patients met the inclusion and exclusion criteria and were clustered into three groups according to the APAR tertiles (T1: ≤ 1.961, T2: 1.961–3.138, T3: > 3.138; Figure 1). The baseline characteristics of individuals are depicted in Table 1. Patients were largely white (65.4%) and male (61.4%), with a median age of 74.4 (IQR, 65.2, 82.4) years. Patients with the highest APAR had more tendency to be older, suffering from heart failure, diabetes, and CKD, and exhibiting higher levels of WBC, platelet, PT, INR, ALT, AST, creatinine, BUN, potassium, lactate, anion gap, and SOFA score, as well as lower hemoglobin, PO_2_, sodium, and BE. Patients in the T3 group had lower use of aspirin, statins, and CCBs. As APAR increased, the 28-day all-cause mortality increased, and the 365-day all-cause mortality showed the same trend. This suggested that APAR was related to poor prognosis in critically ill patients with AF.

### 3.2. Study Outcomes

**Figure 2:**
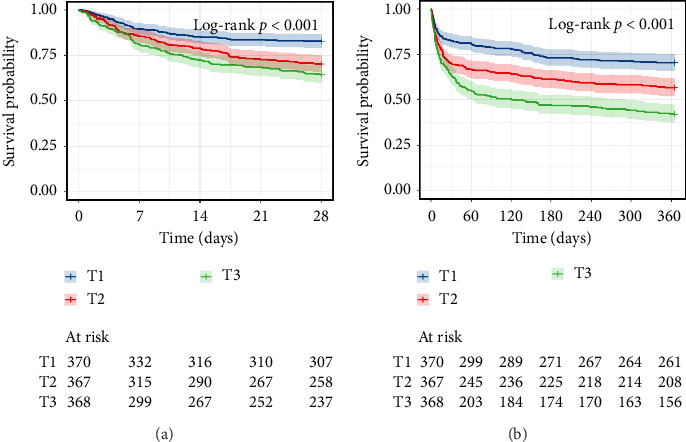
Kaplan–Meier survival curves for all-cause mortality. Kaplan–Meier curves of 28-day (a) and 365-day (b) all-cause mortality stratified by APAR tertiles. APAR, alkaline phosphatase to albumin ratio; APAR tertiles: T1 (≤ 1.961), T2 (1.961–3.138), and T3 (> 3.138).

The Kaplan–Meier curves indicated the differences in the all-cause mortality at 28 days and 365 days among the APAR tertiles (Figure 2). A total of 303 of the 1105 patients died within 28 days, and 481 of the 1105 patients died within 365 days. Patients in the highest APAR group exhibited significantly higher mortality rates both at 28 days and 365 days. Furthermore, the log-rank test demonstrated that the mortality rate significantly elevated as the APAR tertile increased (28-day: *p* < 0.001; 365-day: *p* < 0.001).

### 3.3. Relationship Between APAR and All-Cause Mortality of Patients With AF

To identify the independent effect of APAR on 28-day and 365-day all-cause mortality in patients with AF, multivariate Cox regression models were employed (Table 2).

When APAR was regarded as a continuous variable, Cox proportional hazards analysis suggested that APAR was significantly related to 28-day all-cause mortality both in the unadjusted Model 1 (HR, 1.03 [95% CI 1.02–1.05], *p* < 0.001) and fully adjusted Model 3 (HR, 1.02 [95% CI [1.00–1.04], *p*=0.017). APAR was also associated with 365-day all-cause mortality in Model 1 (HR, 1.03 [95% CI 1.02–1.05], *p* < 0.001) and Model 3 (HR, 1.02 [95% CI 1.01–1.04], *p*=0.004). When APAR was calculated as a nominal variable, the highest APAR was associated with an increased risk of 28-day mortality both in Model 1 (HR, 2.32 [95% CI 1.72–3.14], *p* < 0.001) and Model 3 (HR, 1.64 [95% CI 1.20–2.25], *p*=0.002). A similar pattern could be observed in the relationship between APAR and all-cause mortality at 365 days. The risk of mortality at 28 days and 365 days exhibited an upward tendency as the APAR tertiles increased both in unadjusted and fully adjusted models. To validate the robustness of our findings regarding missing data, we conducted a complete-case analysis (excluding all cases with missing values). Among the 1105 patients, 925 (83.7%) had complete data for all covariates. The complete-case analysis yielded qualitatively consistent results with the imputed dataset. The highest APAR tertile remained significantly associated with 28-day mortality (HR, 1.63 [95% CI 1.18–2.26], *p*=0.003) and 365-day mortality (HR, 1.91 [95% CI 1.48–2.46], *p* < 0.001) in the fully adjusted Model 3.

**Figure 3:**
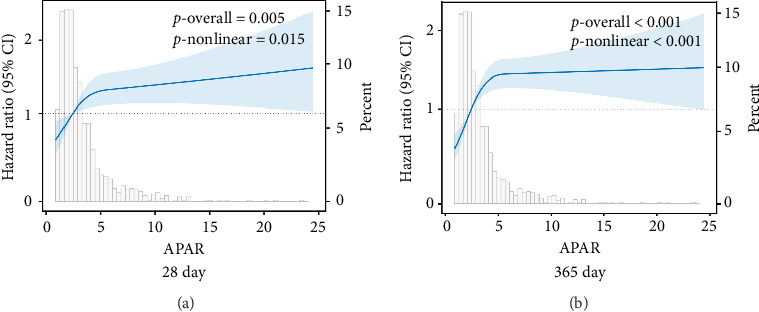
Restricted cubic spline analysis of APAR with all-cause mortality. Restricted cubic spline analysis of BAR with 28-day (a) and 365-day (b) all-cause mortality. APAR, alkaline phosphatase to albumin ratio; CI, confidence interval.

Additionally, the RCS curve analysis indicated the nonlinear association between APAR and all-cause mortality at 28 days and 365 days (28-day: P-nonlinear = 0.015; 365-day: P-nonlinear < 0.001; Figure 3).

**Figure 4:**
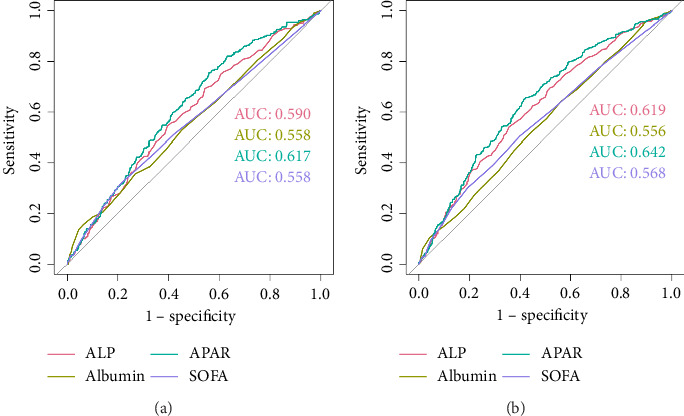
The ROC analysis of all-cause mortality. ROC curves for 28-day (a) and 365-day (b) all-cause mortality. ALP, alkaline phosphatase; APAR, alkaline phosphatase to albumin ratio; AUC, area under the curve; ROC, receiver operating characteristic; SOFA, Sequential Organ Failure Assessment.

To further evaluate the predictive value of APAR for all-cause mortality, the ROC curves were plotted for ALP, albumin, APAR, and SOFA score (Figure 4). The AUC of 28-day mortality for APAR was 0.617 (95% CI 0.581–0.653), which showed statistically significant but modestly higher AUC values compared to ALP (AUC = 0.590, 95% CI 0.553–0.627, *p* < 0.001), albumin (AUC = 0.558, 95% CI 0.520–0.597, *p*=0.009), and SOFA score (AUC = 0.558, 95% CI 0.519–0.596, *p*=0.023). For 365-day all-cause mortality, APAR (AUC = 0.642, 95% CI 0.609–0.674) also showed statistically significant but modestly higher AUC values compared to ALP (AUC = 0.619, 95% CI 0.585–0.652, *p* < 0.001), albumin (AUC = 0.556, 95% CI 0.522–0.590, *p* < 0.001), and SOFA score (AUC = 0.568, 95% CI 0.535–0.602, *p*=0.002). These findings indicated the prognostic value of APAR in predicting AF mortality.

### 3.4. Subgroup Analysis

**Figure 5:**
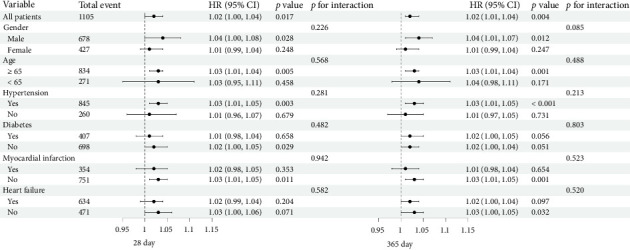
Forest plots of stratified analyses of alkaline phosphatase to albumin ratio and all-cause mortality at 28 days and 365 days. CI, confidence interval; HR, hazard ratio.

Subgroup analysis based on age (< 65 or ≥ 65), sex (male or female), hypertension, diabetes, myocardial infarction, and heart failure was performed to investigate whether APAR remained an independent predictor in different individual subgroups (Figure 5). The HRs for 28-day and 365-day all-cause mortality were significant in subgroups of males and those aged ≥ 65 years. The association between APAR and mortality at 28 days and 365 days was all statistically significant in patients with hypertension and without myocardial infarction. The correlation between APAR and 28-day all-cause mortality was only significant in patients without diabetes. The HR for 365-day all-cause mortality was significant in patients without heart failure. The results did not indicate any significant interactions between APAR levels and different subpopulations.

### 3.5. The Correlation of APAR and WBC, ALT, AST, and Creatinine

Spearman's correlation analysis showed a significant positive correlation between APAR and WBC levels (correlation = 0.1549, *p* < 0.001). APAR also exhibited significant positive correlations with the ALT (correlation = 0.2470, *p* < 0.001), AST (correlation = 0.1536, *p* < 0.001), and creatinine (correlation = 0.2463, *p* < 0.001).

## 4. Discussion

The present study aimed to elucidate the association between APAR and all-cause mortality in critically ill patients with AF. We retrospectively enrolled 1105 AF patients first admitted to the ICU and found that patients with elevated APAR levels had increased all-cause mortality at 28 days and 365 days, even after adjusting for multiple confounding factors. These results suggested that APAR was a simple and readily available biomarker for the identification of AF patients at high risk of mortality. To fully understand the significance of this composite index, it is essential to delineate the distinct pathophysiological roles of its individual components in cardiovascular disease.

ALP is a kind of membrane-binding glycoprotein that catalyzes the hydrolysis of organic pyrophosphate. It is widely expressed in tissues of the human body, especially in the liver, bone, and kidney. Elevated ALP levels are associated with various pathological conditions, particularly those related to bone and liver disease. Recent evidence has implicated the potential role of ALP in promoting vascular calcification and atherosclerosis by catalyzing the hydrolysis of pyrophosphate, which is an inhibitor of vascular calcification [21]. In clinical settings, the relationship between ALP and diabetes, myocardial infarction, ischemic heart disease, and stroke has been well established. A retrospective study indicated that myocardial infarction patients with elevated ALP levels exhibited lower survival rates, and a significant correlation was observed between ALP and peak plasma cardiac troponin I [22]. Another study involving 1426 patients with ischemic heart disease suggested the association between elevated ALP levels and increased risk of 3-year mortality [23]. For patients with stroke, elevated ALP levels were linked to a 2.4-fold increased risk of mortality [21]. The underlying mechanisms might involve several pathways. Firstly, AF patients with elevated ALP levels had a higher probability of vascular calcification and increased stiffness of blood vessel walls. A study evaluated 470 patients with stable angina pectoris and demonstrated that increased ALP levels were positively related to Gensini scores [24]. Secondly, higher ALP levels were associated with impaired renal function and inflammatory activation, both of which were established indicators of poor prognosis in AF patients [23]. In AF patients with heart failure, passive venous congestion might increase the venous pressure of the hepatobiliary system, leading to a significant increase in ALP levels [11]. Elevated serum ALP, rather than ALT or AST, was significantly related to adverse outcomes in heart failure patients without a history of hepatic disease [25]. In addition, previous studies suggested that ALP was a simple biomarker for mortality and recurrent vascular events in patients with stroke [26, 27]. Thus, in AF patients with stroke, those who had elevated ALP levels might have a poor prognosis. Lastly, growing evidence confirmed that metabolic disturbances lead to electrophysiological remodeling in AF [28]. The interference of glycolysis and fatty acid uptake caused by elevated ALP might be another critical factor contributing to adverse outcomes in patients with AF [29, 30]. In our study, the significant positive correlations between APAR and WBC, ALT, AST, and creatinine further supported that APAR reflected a state of inflammation, hepatic insufficiency, and renal dysfunction.

Albumin, the predominant protein within the circulatory system, is responsible for the maintenance of fluid balance and the transportation of various substances [12]. Beyond serving as an indicator of nutritional status and hepatic synthetic function, albumin is intricately involved in the pathophysiology of cardiovascular diseases. A retrospective study of 126 AF patients identified that lower albumin levels were associated with increased previous cardioversions and the enlargement of left atrial volume. Additionally, decreased albumin was an independent predictor of AF recurrence following pulmonary vein isolation ablation [13]. In patients with stable coronary artery disease, decreased albumin levels were also independently related to worse cardiovascular outcomes [31]. There are several intrinsic mechanisms of albumin as a protective factor for AF. Firstly, inflammation and oxidative stress are involved in the progression of AF [32]. Atrial biopsies taken from AF patients exhibited higher levels of inflammatory infiltrates and oxidative injury [33, 34]. It has been confirmed that albumin relieves oxidative stress and inhibits inflammation in AF by scavenging hydroxyl and free radicals [35]. Furthermore, the antiplatelet and anticoagulant activity of albumin may also contribute to its protective role in AF [12]. Therefore, a low albumin level indicates a loss of these beneficial effects.

Considering the pathophysiological implications of elevated ALP and reduced albumin, their combination as APAR offers a composite indicator that provides an integrated assessment of the patient's status. It reflects both an increase in vascular and metabolic dysfunction and a deficit of endogenous protective proteins. This synergistic combination might explain why APAR demonstrated a statistically significant, though modest, improvement in predictive ability compared to either component alone or the SOFA score in our ROC analysis. Moreover, APAR offered a simpler and more convenient alternative for clinicians to evaluate the condition and prognosis of AF patients. This finding was consistent with previous studies that had explored the prognostic value of APAR in various diseases. A study of 390 patients undergoing minimally invasive lung cancer surgery determined that APAR could be a tool to improve prognostic prediction [15]. Another study that enrolled 3378 coronary artery disease patients following percutaneous coronary intervention confirmed the predictive role of APAR for all-cause mortality and cardiac mortality [16]. Additionally, APAR has been linked to adverse clinical outcomes in critically ill patients with sepsis [14]. However, no studies explored the relationship between APAR and prognosis in AF patients. The present study suggested that APAR was associated with all-cause mortality in critically ill patients with AF. Consequently, APAR might facilitate simplified risk stratification and timely adjustment of treatment strategies for patients with severe AF, primarily due to its simplicity and ready availability from routine blood tests.

When considering the prognostic value of APAR, the potential confounding effects of systemic inflammatory response syndrome (SIRS) and malnutrition, both highly prevalent in critically ill populations, should be addressed. SIRS can elevate ALP through cholestatic mechanisms and reduce albumin synthesis due to the hepatic reprioritization of acute-phase proteins. Similarly, malnutrition directly contributes to hypoalbuminemia [36]. Although we adjusted for the SOFA score and key comorbidities, residual confounding from the unmeasured severity of SIRS or nutritional status may persist. The significant correlations between APAR and markers of inflammation and organ dysfunction suggested that APAR captured aspects of this systemic illness burden.

Despite the relatively large sample size, this study has several limitations. Firstly, as a single-center retrospective study, the population selection bias existed. Secondly, the study used the first APAR measurement within 24 h of ICU admission to predict 28-day and 365-day all-cause mortality, without dynamic monitoring of APAR levels. Thirdly, due to the missing data, this study did not include variables like C-reactive protein, cholesterol, and triglyceride, which were also crucial to the prognosis of AF. Despite imputation, the residual bias from missing data could not be entirely ruled out. Finally, due to the nature of the retrospective study, we could not establish the causal relationship between APAR and mortality. The modest AUC of APAR suggested its clinical utility might require integration with other biomarkers for mortality prediction. Multicenter studies were warranted to establish the optimal threshold of APAR in predicting AF mortality. The mechanisms behind the finding need to be further investigated.

## 5. Conclusions

The current study suggested that APAR was an easily accessible biomarker for the prediction of all-cause mortality in AF patients admitted to ICU. Further multicenter and prospective studies are warranted to validate the predictive value of APAR in AF.

## Figures and Tables

**Table 1:** Patient demographics and baseline characteristics.

Characteristic	APAR tertile				*p* value
	Overall (*N* = 1105)	T1 (*N* = 370)	T2 (*N* = 367)	T3 (*N* = 368)	
Age, years	74.4 (65.2, 82.4)	73.9 (64.3, 81.8)	73.1 (64.6, 81.6)	76.2 (66.2, 83.6)	0.039

Male	678 (61.4%)	227 (61.4%)	240 (65.4%)	211 (57.3%)	0.081

Race (white)	723 (65.4%)	236 (63.8%)	246 (67.0%)	241 (65.5%)	0.651

BMI (kg/m^2^)	28.1 (24.3, 33.3)	28.0 (24.2, 32.4)	28.9 (24.8, 34.2)	27.5 (23.9, 32.9)	0.101

*Vital signs*					
SBP (mmHg)	119.0 (103.0, 137.0)	119.0 (103.0, 134.0)	123.0 (106.0, 141.0)	117.0 (100.8, 135.3)	0.023
DBP (mmHg)	65.0 (54.0, 78.0)	63.0 (52.3, 76.0)	66.0 (56.0, 79.5)	65.0 (53.0, 78.0)	0.020
MBP (mmHg)	81.0 (69.0, 94.0)	80.0 (69.0, 93.0)	82.0 (71.0, 97.5)	79.0 (65.8, 93.0)	0.009
HR (beats/min)	89.0 (75.0, 106.0)	85.0 (74.0, 100.8)	90.0 (78.0, 108.0)	92.0 (76.0, 110.0)	< 0.001
RR (times/min)	19.0 (16.0, 24.0)	18.0 (15.0, 22.0)	20.0 (16.0, 24.0)	20.0 (16.0, 24.0)	< 0.001
Temperature (°C)	36.7 (36.3, 37.1)	36.6 (36.3, 36.9)	36.8 (36.4, 37.2)	36.7 (36.3, 37.1)	< 0.001

*Laboratory results*					
WBC (K/μL)	12.1 (8.3, 17.3)	11.2 (7.7, 15.9)	11.8 (8.6, 17.4)	13.6 (9.0, 19.8)	< 0.001
RBC (m/μL)	3.6 (3.1, 4.2)	3.7 (3.0, 4.2)	3.7 (3.2, 4.2)	3.5 (3.1, 4.0)	0.011
Platelet (K/μL)	190.0 (138.0, 259.0)	177.0 (125.3, 234.0)	186.0 (146.0, 254.0)	213.0 (147.0, 288.0)	< 0.001
Hemoglobin (g/dL)	10.8 (9.1, 12.4)	11.2 (9.2, 12.7)	10.9 (9.4, 12.5)	10.2 (9.0, 11.8)	< 0.001
PT (s)	15.3 (13.2, 20.2)	14.9 (12.9, 18.6)	14.9 (13.1, 19.5)	16.2 (13.7, 23.0)	< 0.001
PTT (s)	33.2 (28.2, 48.4)	33.2 (28.1, 53.1)	31.9 (27.9, 43.4)	34.2 (29.1, 48.6)	0.067
INR	1.4 (1.2, 1.8)	1.4 (1.2, 1.7)	1.4 (1.2, 1.8)	1.5 (1.2, 2.1)	< 0.001
PO_2_ (mmHg)	97.0 (56.0, 217.0)	127.5 (73.0, 321.0)	90.0 (54.5, 191.0)	83.5 (47.0, 144.3)	< 0.001
ALT (IU/L)	30.0 (17.0, 73.0)	22.0 (15.0, 43.0)	30.0 (17.0, 71.5)	45.5 (22.0, 122.3)	< 0.001
AST (IU/L)	45.0 (26.0, 121.0)	39.0 (25.0, 76.8)	41.0 (24.5, 116.0)	61.0 (30.8, 169.0)	< 0.001
Creatinine (mg/dL)	1.3 (0.9, 2.2)	1.1 (0.8, 1.7)	1.4 (0.9, 2.2)	1.6 (1.1, 2.7)	< 0.001
BUN (mg/dL)	28.0 (19.0, 47.0)	22.5 (16.0, 36.0)	28.0 (18.0, 44.0)	37.0 (23.0, 58.0)	< 0.001
Sodium (mEq/L)	139.0 (136.0, 142.0)	139.0 (137.0, 142.0)	139.0 (136.0, 142.0)	138.0 (134.0, 141.0)	< 0.001
Potassium (mEq/L)	4.2 (3.8, 4.7)	4.2 (3.8, 4.7)	4.2 (3.8, 4.7)	4.4 (3.8, 4.9)	0.037
Albumin (g/L)	31.0 (27.0, 35.0)	33.0 (29.0, 37.0)	32.0 (28.0, 35.0)	28.0 (24.0, 32.0)	< 0.001
Lactate (mmol/L)	1.7 (1.2, 2.7)	1.6 (1.2, 2.3)	1.7 (1.2, 2.8)	1.9 (1.3, 3.1)	< 0.001
Glucose (mg/dL)	142.0 (111.0, 186.0)	141.0 (115.0, 188.0)	140.0 (113.5, 180.5)	144.5 (105.0, 188.5)	0.568
Anion gap (mEq/L)	16.0 (13.0, 19.0)	14.0 (13.0, 17.0)	16.0 (14.0, 19.0)	17.0 (14.0, 20.0)	< 0.001
BE (mmol/L)	−1.0 (−5.0, 1.0)	0.0 (−4.0, 2.0)	−1.0 (−5.0, 1.0)	−2.0 (−6.0, 0.0)	< 0.001
ALP (IU/L)	77.0 (55.0, 109.0)	49.0 (40.0, 58.8)	77.0 (65.5, 90.0)	129.0 (106.0, 180.0)	< 0.001
APAR	2.4 (1.7, 3.7)	1.6 (1.3, 1.7)	2.4 (2.2, 2.8)	4.5 (3.7, 6.8)	< 0.001

*Score system*					
SOFA	2.0 (0.0, 4.0)	2.0 (0.0, 5.0)	2.0 (0.0, 4.0)	3.0 (1.0, 5.0)	0.005

*Comorbid disease*					
Myocardial infarction	354 (32.0%)	112 (30.3%)	127 (34.6%)	115 (31.3%)	0.418
Heart failure	634 (57.4%)	187 (50.5%)	223 (60.8%)	224 (60.9%)	0.005
PVD	220 (19.9%)	92 (24.9%)	66 (18.0%)	62 (16.8%)	0.013
CVD	200 (18.1%)	75 (20.3%)	62 (16.9%)	63 (17.1%)	0.412
Hypertension	845 (76.5%)	289 (78.1%)	281 (76.6%)	275 (74.7%)	0.556
Diabetes	407 (36.8%)	109 (29.5%)	134 (36.5%)	164 (44.6%)	< 0.001
CKD	369 (33.4%)	85 (23.0%)	119 (32.4%)	165 (44.8%)	< 0.001

*Medication*					
Aspirin	579 (52.4%)	216 (58.4%)	196 (53.4%)	167 (45.4%)	0.002
Clopidogrel	109 (9.9%)	35 (9.5%)	41 (11.2%)	33 (9.0%)	0.575
Statin	483 (43.7%)	177 (47.8%)	173 (47.1%)	133 (36.1%)	0.002
Amiodarone	410 (37.1%)	150 (40.5%)	128 (34.9%)	132 (35.9%)	0.235
CCB	146 (13.2%)	72 (19.5%)	46 (12.5%)	28 (7.6%)	< 0.001
ACEI/ARB	203 (18.4%)	70 (18.9%)	68 (18.5%)	65 (17.7%)	0.903
Warfarin	242 (21.9%)	92 (24.9%)	78 (21.3%)	72 (19.6%)	0.206
NOACs	72 (6.5%)	19 (5.1%)	37 (10.1%)	16 (4.3%)	0.003

*Outcome*					
28-day mortality	303 (27.4%)	63 (17.0%)	109 (29.7%)	131 (35.6%)	< 0.001
365-day mortality	481 (43.5%)	109 (29.5%)	159 (43.3%)	213 (57.9%)	< 0.001

*Note:* APAR tertiles: T1 (≤ 1.961), T2 (1.961–3.138), and T3 (> 3.138).

Abbreviations: ACEI/ARB, angiotensin-converting enzyme inhibitors or angiotensin receptor blockers; ALP, alkaline phosphatase; ALT, alanine aminotransferase; APAR, alkaline phosphatase to albumin ratio; AST, aspartate aminotransferase; BE, base excess; BMI, body mass index; BUN, blood urea nitrogen; CCBs, calcium channel blockers; CKD, chronic kidney disease; CVD, cerebrovascular disease; DBP, diastolic blood pressure; HR, heart rate; INR, international normalized ratio; MBP, mean blood pressure; NOACs, nonvitamin K antagonist oral anticoagulants; PO_2_, partial pressure of O_2_; PT, prothrombin time; PTT, partial prothrombin time; PVD, peripheral vascular disease; RBC, red blood cell; RR, respiratory rate; SBP, systolic blood pressure; SOFA, Sequential Organ Failure Assessment; WBC, white blood cell.

**Table 2:** Cox analysis of APAR for 28-day and 365-day all-cause mortality.

Exposure	Model 1		Model 2		Model 3	
APAR	HR (95% CI)	*p* value	HR (95% CI)	*p* value	HR (95% CI)	*p* value
*28-day mortality*						
Continuous variable per unit	1.03 (1.02–1.05)	< 0.001	1.03 (1.02–1.05)	< 0.001	1.02 (1.00–1.04)	0.017
T1 (*N* = 370)	Ref		Ref		Ref	
T2 (*N* = 367)	1.85 (1.36–2.53)	< 0.001	1.85 (1.35–2.52)	< 0.001	1.73 (1.26–2.37)	< 0.001
T3 (*N* = 368)	2.32 (1.72–3.14)	< 0.001	2.21 (1.64–2.99)	< 0.001	1.64 (1.20–2.25)	0.002
*p* for trend		< 0.001		< 0.001		0.004

*365-day mortality*						
Continuous variable per unit	1.03 (1.02–1.05)	< 0.001	1.03 (1.02–1.05)	< 0.001	1.02 (1.01–1.04)	0.004
T1 (*N* = 370)	Ref		Ref		Ref	
T2 (*N* = 367)	1.64 (1.29–2.10)	< 0.001	1.67 (1.30–2.13)	< 0.001	1.61 (1.25–2.06)	< 0.001
T3 (*N* = 368)	2.44 (1.94–3.08)	< 0.001	2.35 (1.86–2.96)	< 0.001	1.87 (1.47–2.39)	< 0.001
*p* for trend		< 0.001		< 0.001		< 0.001

*Note:* APAR tertiles: T1 (≤ 1.961), T2 (1.961–3.138), and T3 (> 3.138). Model 1: unadjusted model. Model 2: adjusted for male, age, race, and body mass index. Model 3: adjusted for male, age, race, body mass index, heart failure, cerebrovascular disease, diabetes, chronic kidney disease, blood urea nitrogen, lactate, potassium, Sequential Organ Failure Assessment, heart rate, angiotensin-converting enzyme inhibitors or angiotensin receptor blockers, warfarin, and nonvitamin K antagonist oral anticoagulants.

Abbreviations: APAR, alkaline phosphatase to albumin ratio; CI, confidence interval; HR, hazard ratio.

## Data Availability

The data that support the findings of this study are available on request from the corresponding author. The data are not publicly available due to privacy or ethical restrictions.

## Supporting Information

Table S1: Missing percentage of variables.

Table S2: Variance inflation factor between variables.
