# Supplementary material for: Alkaline Phosphatase to Albumin Ratio as a Novel Predictor of All-Cause Mortality in Critically Ill Patients With Atrial Fibrillation
Source: Cardiol Res Pract. 2025 Nov 4;2025:1283547. doi: 10.1155/crp/1283547 (PMC12614730; doi:10.1155/crp/1283547)
Supplement: Supporting Information — Additional supporting information can be found online in the Supporting Information section. [file 1283547.f1.docx]

**Table S1 Missing percentage of variables**

| Variables | Missing percentage (%) | Variables | Missing percentage (%) |
| --- | --- | --- | --- |
| Age | 0 | INR | 2.2 |
| Height | 0 | PO_2_ | 0 |
| Weight | 0 | ALT | 0.6 |
| SBP | 0.5 | AST | 0.2 |
| DBP | 0.5 | Creatinine | 0 |
| MBP | 0 | BUN | 0 |
| HR | 0 | Sodium | 0 |
| RR | 0 | Potassium | 0 |
| Temperature | 6.5 | Albumin | 0 |
| C-reactive protein | 97.3 | Lactate | 6.7 |
| WBC | 0.1 | Glucose | 0 |
| RBC | 0.1 | Anion gap | 0 |
| Platelet | 0.1 | BE | 0 |
| Hemoglobin | 0.1 | ALP | 0 |
| PT | 2.2 | Triglyceride | 84.9 |
| PTT | 2.4 | Cholesterol | 90.6 |
| SOFA | 0 |  |  |

ALP, alkaline phosphatase; ALT, alanine aminotransferase; AST, aspartate aminotransferase; BE, base excess; BUN, blood urea nitrogen; DBP, diastolic blood pressure; HR, heart rate; INR, international normalized ratio; MBP, mean blood pressure; PO_2_, partial pressure of O_2_; PT, prothrombin time; PTT, partial prothrombin time; RBC, red blood cell; RR, respiratory rate; SBP, systolic blood pressure; SOFA, sequential organ failure assessment; WBC, white blood cell.

**Table S2 Variance inflation factor between variables**

| Variable | VIF | Variable | VIF |
| --- | --- | --- | --- |
| Gender | 1.178 | ALT | 6.963 |
| Age | 1.472 | AST | 7.089 |
| Race | 1.041 | Hemoglobin | 7.107 |
| HR | 1.407 | Platelet | 1.288 |
| RR | 1.274 | RBC | 6.864 |
| MBP | 5.227 | WBC | 1.222 |
| Temperature | 1.266 | Lactate | 1.924 |
| SBP | 2.24 | PO_2_ | 1.329 |
| DBP | 4.093 | BE | 1.702 |
| INR | 10.865 | Myocardial infarction | 1.249 |
| PT | 11.1 | Heart failure | 1.252 |
| PTT | 1.238 | PVD | 1.138 |
| Anion gap | 2.119 | CVD | 1.085 |
| Potassium | 1.298 | Hypertension | 1.191 |
| Sodium | 1.108 | Diabetes | 1.293 |
| BUN | 2.163 | SOFA | 1.305 |
| Glucose | 1.251 | Aspirin | 1.343 |
| Creatinine | 2.212 | Clopidogrel | 1.221 |
| Statin | 1.383 | Warfarin | 1.144 |
| Amiodarone | 1.121 | NOACs | 1.096 |
| CCB | 1.107 | CKD | 1.573 |
| ACEI/ARB | 1.129 | BMI | 1.232 |

ACEI/ARB, angiotensin-converting enzyme inhibitors or angiotensin receptor blockers; ALT, alanine aminotransferase; AST, aspartate aminotransferase; BE, base excess; BMI, body mass index; BUN, blood urea nitrogen; CCB, calcium channel blockers; CKD, chronic kidney disease; CVD, cerebrovascular disease; DBP, diastolic blood pressure; HR, heart rate; INR, international normalized ratio; MBP, mean blood pressure; NOACs, non-vitamin K antagonist oral anticoagulants; PO_2_, partial pressure of O_2_; PT, prothrombin time; PTT, partial prothrombin time; PVD, peripheral vascular disease; RBC, red blood cell; RR, respiratory rate; SBP, systolic blood pressure; SOFA, sequential organ failure assessment; WBC, white blood cell.
